# Insulin initiation: bringing objectivity to choice

**DOI:** 10.1186/s40200-015-0146-1

**Published:** 2015-03-25

**Authors:** Sanjay Kalra, Yashdeep Gupta

**Affiliations:** Bharti Hospital, Kunjpura Road, Karnal, 132001 Haryana India; Department of Medicine, Government Medical College and Hospital, Sector 32, Chandigarh, 160030 India

**Keywords:** Basal insulin, Basal-bolus insulin, Diabetes, Insulin, Intensive insulin, Prandial insulin, Premixed insulin

## Abstract

The choice of initial insulin is often dictated by subjective criteria: the “severity” of diabetes, the ability of the person with diabetes to self inject, at specific times of the day, and the physician’s personal experience. No objective criteria have been evolved by any expert body so far to help guide clinicians make an appropriate, and accurate, choice of initiating insulin. Neither have large studies been able to shed light on the preferred type of insulin regime for a particular individual.

This communication suggests various objective parameters which may be used to inform this decision.

## Introduction

The appropriate choice of an insulin regime for initiation of therapy has always been a matter of debate. There is no universal consensus for the optimal method of starting insulin therapy in patients with type 2 diabetes (T2DM) who do not respond to oral anti-diabetic drugs (OADs). While the American Diabetes Association (ADA) and European Association for study of Diabetes (EASD) suggest basal insulin as an initial preferred strategy [1], the International Diabetes Federation (IDF) recommends both premixed and basal insulin [2]. Many national guidelines, on the other hand, support the use of premixed insulin as a preferred choice for initiation of therapy [3]. Intensive insulin therapy, too, is indicated as initial line of management in a select group of patients [1].

### Postprandial glycemia

Much discussion about the relative merits and demerits of these approaches revolves around the relevance of postprandial hyperglycemia. The importance of this entity in diabetes is beyond doubt. It is proven to be associated, independently, with risk of micro vascular complication, macro vascular complications, and mortality. These effects are mediated by a range of adverse biochemical and cellular effect, including oxidative stress, dyslipidemia, insulin resistance, altered blood coagulation, endothelial dysfunction, and increased intima media thickness [4]. The IDF, therefore, clearly recommends that treatment strategies which lower postprandial glycemia must be implemented in those with postprandial hyperglycemia [5].

This is especially important for Asian populations, which experience a greater contribution from postprandial hyperglycemia to overall HbA1c [6]. These cohorts are at risk of greater cardiovascular and all-cause mortality risk with high postprandial glucose values than with high fasting glucose levels [7-9]. Much of the discussion about the appropriateness of basal insulin, therefore, takes on ethnic tones [10,11]. This is unfortunate, as both basal and premixed insulin have a definite role to play in the management of diabetes, in all ethnic groups.

### Subjectivity vs. objectivity

The choice of initial insulin is often dictated by subjective criteria: the “severity” of diabetes, the ability of the person with diabetes to self inject, at specific times of the day, and the physician’s personal experience. No objective criteria have been evolved by any expert body so far to help guide clinicians make an appropriate, and accurate, choice of initiating insulin. Neither have large studies been able to shed light on the preferred type of insulin regime for a particular individual [4-7,12].

This communication suggests various objective parameters which may be used to inform this decision. Shared decision-making, involving patient and physician, is always welcome. These objective criteria, based upon simple physiologic principles, will help characterize the “hyperglycemia personality”, for both. This is important for health care professionals, who confront a wide spectrum of diabetes in clinical practice including predominant fasting hyperglycemia, predominant postprandial hyperglycemia, and overall hyperglycemia. It is also relevant for patients, who would like to understand the rationale behind particular regime.

It must be noted that many of the suggestions listed below are empirical or experience-based, and still need to be supported by evidence. However, they all conform to the school of science known as ‘logical empiricism’. They are built upon sound logic, and supported by extensive observation. All these suggestions are easy to follow at primary care level, and may help improve the quality of care of people with diabetes. Choice of insulin therapy is dictated by three parameters: efficacy, safety, and tolerability. This review aims to help the physician choose an appropriate insulin regimen, which maintains safety and tolerability, without compromising efficacy.

## Short term glycemic indices

### Parameter 1: fasting plasma glucose

Fasting plasma glucose (FPG) is a function of hepatic glucose output, and is a prime target for effective anti-diabetes therapy [1]. Basal insulin is more effective than prandial insulin at controlling fasting glycemia [12]. This observation is concordant with the basic pharmacology of basal insulin.

Fasting plasma glucose levels, therefore, can be considered as an objective marker to guide the choice of insulin preparation. High FPG levels should prompt a basal insulin prescription, while relatively normal FPG levels in the setting of uncontrolled HbA1c may encourage a consideration of alternative regimes.

The drawback with using FPG as a single deciding factor, however, is that it does not assess the relative contribution of postprandial hyperglycemia. This value alone does not help one decide whether to choose a basal or a basal-bolus regime for management of diabetes. Hence, while it should be one of the factors used to inform insulin decision making, it cannot be the sole marker for this purpose.

### Parameter 2: postprandial glucose

Post prandial glucose (PPG) values represent the prandial component of hyperglycemia. Regular self monitoring of glucose allows one to assess PPG at various times of the day. The IDF recommends measurement of PPG 1–2 hours after a meal, using self monitoring of blood glucose (SMBG) [5].

High PPG values imply the need for a rapid acting insulin, either as part of a premixed regime, as prandial regime, or as part of a basal-bolus regime. Relative PPG peaks after each meal may help decide the timing of injections as well. For example, once daily premixed insulin may be administered with the meal that leads to the highest PPG value. Similarly, the timing of the bolus component of a basal plus regime can be decided accordingly.

Postprandial plasma glucose alone, if viewed in isolation, however, suffers the same limitation that FPG does. As an isolated factor to help insulin decision making, PPG does not take the contribution of FPG into account. There should be, for example, a difference in the therapeutic strategy for a person with a PPG of 400 mg/dl and a FPG of 200 mg/dl as compared to another with PPG of 400 mg/dl and FPG of 300 mg/dl. PPG data is also limited by lack of uniformity and standardization, as well as absence of consensus related to timing of assessment, size and quality of meals. Post prandial excursions may also be relatively less in the background of already high fasting glucose values. Hence, PPG cannot be used as a sole factor to drive choice of insulin therapy.

### Parameter 3: post challenge/post load plasma glucose

The post-challenge or post-load plasma glucose is taken as a surrogate marker for prandial hyperglycemia. These values are an improvement upon postprandial glucose values, as they are relatively more standardized and reproducible. However the concept of doing post-challenge plasma glucose values in persons with known diabetes is open to criticism: an artificially induced, invasive, time-consuming procedure which puts the person at risk of (albeit, temporary) hyperglycemia does not make good clinical sense.

Mixed meal tolerance tests have been devised to overcome the issue of an artificial load, but these too, are not appropriate for routine clinical practice [13]. The use of post challenge or post load glucose levels to inform the choice of insulin therapy, therefore, cannot be supported.

### Parameter 4: postprandial glucose excursion

A simple objective way to measure the relative contribution of postprandial glucose excursion (PPGE) [14]. Subtracting FPG from PPG provides information about the need for rapid acting insulin (again, as premixed, as prandial, or as basal- bolus). Table 1 provides an empirical clinical decision aid to decide insulin regimes based upon a combination of FPG, PPG and PPGE.

**Table 1:** **Aid for Insulin Decision (AID)-1**

**Postprandial glucose excursion (mg%)**	**Choice 1**	**Choice 2**
>74 mg%	Rapid acting insulin	Premixed Insulin (50:50)
40-74 mg%	Premixed insulin (30:70)	Premixed Insulin (50:50)
<40 mg%	Basal insulin	Premixed insulin (30:70)

The value 74 mg% is taken by calculating PPGE for the diagnostic cut offs for diabetes (200 mg% and 126 mg%).

The value 40 mg% is taken by calculating PPGE for the diagnostic cut offs for prediabetic (impaired glucose tolerance 140 mg%, and impaired fasting glucose 100 mg%).

The use of PPGE, however, does not take the absolute value of FPG into account. Hence, using PPGE as the only criteria for therapeutic choice may underestimate the need for basal insulin. PPGE, therefore, through a simple decision making aid, must be supplemented by other objective parameters in order to achieve optimal usefulness.

### Parameter 5: prandial: fasting index - postprandial glucose excursion, as a function of fasting plasma glucose

We have discussed the limitation of using FPG, PPG and PPGE as sole drivers for decision making. Combining the three objective parameters into a prandial: fasting index (PFI) obviates some of the concerns discussed above. We therefore propose the following index:

$$ \mathbf{Prandial}:\ \mathbf{fasting}\ \mathbf{index}\ \left(\mathbf{P}\mathbf{F}\mathbf{I}\right) = \left(\mathbf{P}\mathbf{P}\mathbf{G}\mathbf{\hbox{-}}\mathbf{F}\mathbf{P}\mathbf{G}\right)\ /\mathbf{F}\mathbf{P}\mathbf{G} $$

The PFI utilizes PPGE (PPG-FPG) as a marker of prandial hyperglycemia, and FPG as a representative of fasting hyperglycemia. A high ratio implies a higher prandial component, which will require premixed or rapid acting insulin for addressal. A low ratio suggests a greater contribution of the fasting component of hyperglycemia, and supports the use of basal insulin.

While the use of this ratio as an aid in insulin decision making has not been analyzed in a research setting so far, Table 2 suggests some arbitrary cut offs to determine choice of insulin regimes. The PFI is limited by the fact that it does not convey the ‘severity’ of hyperglycemia. For example, values of FPG 200 mg/dl, PPG 300 mg/dl give a PFI of 300-200/200 = 0.5, while results of FPG 100 mg/dl, and PPG 150 mg/dl also lead to a PFI of 150-100/100 = 0.5. Thus using PFI alone may not help the treating physician sense the gravity of a particular clinical situation, and may lead to inappropriate choices, especially between basal and basal-bolus regimes.

**Table 2:** **Aid for Insulin Decision (AID)-2**

**PFI =** **(** **PPG-FPG)/FPG**	**Choice 1**	**Choice 2**
>0.6	Rapid acting insulin	Premixed insulin (50:50)
0.4-0.6	Premixed insulin (30:70)	Premixed insulin (50:50)
<0.4	Basal insulin	Premixed insulin (30:70)

The value 0.6 is taken by calculating PFI for the diagnostic cut offs of diabetes (200 mg%, 126 mg%).

The value 0.4 is taken by calculating PFI for the diagnostic cut offs of prediabetes.

## Long term glycemic indices

### Parameter 6: glycated hemoglobin (HbA1c)

The limitation of PFI, discussed in the preceding section, can be overcome by using HbA1c to provide an accurate idea of the severity of diabetes. As a single parameter, however, HbA1c is unable to convey the relative contributions of fasting and postprandial hyperglycemia. There is robust data to suggest that fasting glycemia contributes to the bulk of hyperglycemic burden at high HbA1c [15], and postprandial glycemia at lower HbA1c levels. This however, does not mean that patients with high HbA1c should be managed with basal insulin alone, while those with relatively lower values should receive rapid acting insulin. Empirically, in fact, basal- bolus regimes are chosen to manage ‘very high’ HbA1c levels, while lesser dose regimes are able to suffice for patients with ‘less high’ HbA1c.

### Parameter 7: fasting plasma glucose (FPG): glycated hemoglobin (HbA1c) ratio

The limitations discussed for objective parameters based upon overall status of glycemia (HbA1c) and specific components of glycemia (FPG, PPG) can be overcome if both are combined in one ratio. This has been studied by Vahatalo M et al. [16,17]. The ratio FPG (mmol/l):HbA1c(%) was used to decide the relative contribution of fasting glycemia. A cut off of 1.3 was taken, based upon the diagnostic limit for diabetes based upon FPG (7.8 mmol/l) and HbA1c (6.0%) [17].

The authors, studying type 2 diabetes patients in a randomized controlled trial setting, compared once daily and twice daily intermediate acting insulin (neutral protamine Hagedorn or Lente insulin), with or without oral drug [17]. A ratio of ≥ 1.3 (found in 60% subjects), which suggested fasting hyperglycemia predominance, responded equally to all four regimes studied. Persons with a low ratio (<1.3), suggestive of overall hyperglycemia, responded better to twice daily insulin.

The same authors observed that ‘fasting type’ hyperglycemia is associated with greater body mass index, higher serum triglyceride, hs-CRP and ALT levels at baseline, and more weight gain after treatment with insulin. The weight gain was not dependent on type of basal insulin used. These observations suggest that ‘fasting type’ hyperglycemia is a presentation of greater insulin resistance. This hypothesis was corroborated by the fact that this cohort had a significantly higher daily insulin requirement (0.77 IU/kg/day) than those with ‘overall hyperglycemia’ (0.57 IU/kg/day) [16].

While this study has not compared basal-bolus and premixed regimes, its results can be used to create an empirical tool for decision making (Table 3). This, too, will have to be validated by research on a large scale. A limiting factor, however, is that it does not indicate the absolute severity of the hyperglycemia burden, as mentioned for the PFI.

**Table 3:** **Aid for Insulin Decision (AID)-3**

**FPG/HbA1c**	**Rationale**	**Choice 1**	**Choice 2**
≥ 1.3* (≥20**)	Higher ratio means fasting type hyperglycemia	Basal insulin	Premixed insulin with dinner
≤1.3* (≤20**)	Lower ratio means postprandial type hyperglycemia	Premixed insulin	Premixed insulin with breakfast

*The value 1.3 is calculated with the diagnostic cut offs of diabetes using FPG (7.8 mmol/l) and HbA1c (6%).

**The value 20 is calculated with the diagnostic cut offs for diabetes using FPG (126 mg%) and currently accepted HbA1c (6.3%) levels.

### Parameter 8: anhydroglucitol

1,5-Anhydroglucitol (1,5-AG) is a six-carbon chain monosaccharide and the component of normal human blood serum. It was first discovered in the plant Polygala senega in 1888. It is closely correlated with glycometabolism. In 2003, US FDA approved it as a short-term marker of glycemic control. Serum 1,5-anhydroglucitol (1,5-AG) drops as serum glucose rises above the renal threshold for glucose and has been proposed as a marker for postprandial hyperglycemia.

In clinical practice, A1C and 1,5-AG may be used sequentially, first utilizing the A1C assay to identify patients who are moderately or well controlled (A1C 6.5–8.0%) and then using the 1,5-AG assay to determine the extent of postprandial glucose excursions. If the A1C is above target and the 1,5-AG is normal, this would mean that postprandial glucose excursions are not high and the therapy targeting basal glucose may be more useful. On the other hand, if the A1C is above target and the 1,5-AG is low, would reflect postprandial glucose elevations [18].

So, a low anhydroglucitol value will imply the need for an insulin regime which includes a prandial component, ie, rapid acting insulin or premixed insulin, Such a value may encourage use of a 50:50 biphasic rather than a 30:70 or 25:75 biphasic insulin (with 25 or 30% being proportion of rapid acting insulin). Anhydroglucitol, however, is not available for use in routine clinical practice, and its cost may limit its utility [19].

If anhydroglucitol estimation were to become readily available, a simple method of assessing relative postprandial contribution to hyperglycemia would be to calculate the anhydroglucitol: HbA1c ratio. This may be a more effective method of deciding insulin therapy as compared to a single value of anhydroglucitol.

## Conclusion

Choosing an appropriate regime for insulin initiation is a difficult task. The large variety of options available to us, with an equally large list of supporting evidences in published literature, reinforce the fact that there is no single answer to this clinical challenge. The current status of our knowledge leads to confusion amongst health care providers, many of whom practice a “one approach fits all” approach, based on their training and experience. Such an approach is suboptimal, keeping in mind the wide diversity of diabetes.

There is a need to develop simple, yet accurate, objective tools to aid in decision making. These tools should be usable at primary care level, where the vast majority of people with diabetes take treatment. They should empower physicians and people with diabetes to take appropriate decision, instead of confusing them further. This communication has tried to highlight a few such objective tools. The prandial fasting index (FPI) and the FPG/ HbA1c ratio are two promising aids to insulin decision making, which must be studied further in research as well as clinical settings. The merits and demerits of three easy- to- use indices, using freely available glycemic parameters, are summarized in Table 4.

**Table 4:** **Merits and Demerits of 3 indices, calculated using freely available glycemic data**

**Index/features**	**Postprandial glucose excursion**	**Prandial: fasting index**	**Fasting plasma glucose (FPG): glycated hemoglobin (HbA1c) ratio**
Formula	PPBG-FPG	Prandial: fasting index (PFI) = (PPG-FPG) /FPG	FPG:HbA1c
Need for HbA1c	No	No	Yes
Need for postprandial blood glucose	Yes	Yes	No
Potential for day to day variability	High	High	Low
Ability to decide need for prandial coverage	Good	Good	Relatively less
Ability to decide need for fasting coverage	Relatively less	Fair	Good
Economy	Low cost	Low cost	High cost due to HbA1c
Ability to calculate based on self monitoring of blood glucose	Yes	Yes	No

FPG: Fasting plasma glucose; PPBG: Postprandial blood glucose.
